# Prelacteal feeding practices in Vietnam: challenges and associated factors

**DOI:** 10.1186/1471-2458-13-932

**Published:** 2013-10-07

**Authors:** Phuong H Nguyen, Sarah C Keithly, Nam T Nguyen, Tuan T Nguyen, Lan M Tran, Nemat Hajeebhoy

**Affiliations:** 1 International Food Policy Research Institute, Hanoi, Vietnam; 2 Institute of Social and Medical Studies, Hanoi, Vietnam; 3 FHI 360, Hanoi, Vietnam

**Keywords:** Prelacteal feeding, Breastfeeding, Infant and young child feeding practices, Behavioral determinants, Vietnam

## Abstract

**Background:**

Despite the importance of early initiation of and exclusive breastfeeding, prelacteal feeds continue to pose a barrier to optimal breastfeeding practices in several countries, including Vietnam. This study examined the factors associated with prelacteal feeding among Vietnamese mothers.

**Methods:**

Data from 6068 mother-child (<6 m) dyads were obtained from a cross-sectional survey conducted in 11 provinces in Vietnam in 2011. Multivariate logistic regression analyses were used to examine factors associated with prelacteal feeding.

**Results:**

During the first three days after birth, 73.3% of the newborns were fed prelacteals, 53.5% were fed infants formula, and 44.1% were fed water. The odds of feeding prelacteals declined with increased breastfeeding knowledge, beliefs about social norms in favor of exclusive breastfeeding, and confidence in one’s own breastfeeding behaviors. Women who harbored misconceptions about breastfeeding had twice the odds of feeding any prelacteals (OR: 2.09, 95% CI: 1.74–2.50). Health care factors increasing the odds of prelacteal feeding included delivery by caesarean section (OR: 2.94, 95% CI: 2.39–3.61) or episiotomy (OR: 1.36, 95% CI: 1.17–1.58) and experiencing breastfeeding problems (OR: 1.31, 95% CI: 1.04–1.66). Health staff support during pregnancy and after birth reduced the odds of feeding formula. However, family support after delivery increased the odds of feeding water to newborns.

**Conclusions:**

The multiple factors contributing to the high prevalence of prelacteal feeding behaviors stress the need for early and appropriate breastfeeding interventions in Vietnam, particularly during routine healthcare contacts. Improving breastfeeding practices during the first days of an infant’s life could be achieved by improving knowledge and confidence of mothers through appropriate perinatal counseling and support. Ensuring that health facilities integrate these practices into routine ante-natal care and post-delivery management is critical.

## Background

Vietnam has made great strides in child nutrition over the past decade, achieving declines in the prevalence of underweight and stunting by roughly 20% and 10%, respectively [1]. These gains, however, have not kept pace with economic development and have lagged behind progress observed in other health indicators such as maternal and infant mortality, access to clean water, and basic sanitation [2,3]. Undernutrition remains a public health concern in Vietnam, where 29.3% of children under five are stunted and 17.5% are underweight [1]. Estimates from a 2010 national surveillance study reveal that out of 63 provinces, 37 had a high or very high prevalence of stunting (≥30%), and 27 had high rates of underweight (20-29%) [1]. Populations with elevated rates of undernutrition face numerous health and economic consequences, including greater risk of childhood morbidity and mortality, susceptibility to chronic diseases in adulthood, impaired cognitive development, and reduced economic productivity [4,5].

Practicing optimal breastfeeding is one of the most effective and cost-efficient ways to prevent undernutrition [6]. Although breastfeeding is the norm in Vietnam, practiced by 98% of mothers [7], breastfeeding behaviors remain far from ideal. One-third of infants do not receive breastmilk within an hour of birth, and only one-fifth are exclusively breastfed for the first six months of life [1]. Evidence suggests that prelacteal feeding – giving foods or liquids to newborns before breastfeeding is established or before breastmilk ‘comes in’ [8-11]– is a major contributor to suboptimal breastfeeding patterns in Vietnam [12,13]. Giving prelacteals has been shown to delay the initiation of breastfeeding [9] and interfere with exclusive breastfeeding (EBF) during the first six months of life [8,14-16]. The relationship between prelacteal feeding and breastfeeding is often described as a ‘vicious cycle’ [17,18]. By reducing the amount of time an infant suckles at the breast, prelacteal feeding can lead to delayed lactation and reduced breastmilk supply, both of which encourage mothers to continue feeding prelacteals [17].

Prelacteal feeding has been linked with negative neonatal health outcomes, including increased risk of illness [16,19] and possibly even mortality [20]. Colostrum, or the ‘first milk’ produced in the first three days following delivery, contains a high proportion of immunological agents [21]. By interfering with breastfeeding during this period, prelacteal feeding diminishes the immunological benefits a newborn receives, thus increasing his/her susceptibility to infection. In addition, prelacteal feeding can be a direct cause of illness by exposing infants to contaminated feeds, utensils, water, or hands. Prelacteal feeding may also affect neonatal health by disrupting the priming of the gastrointestinal tract [22]. Further, mother-infant attachment may be interrupted by prelacteal feeding as it reduces close skin-to-skin contact between a mother and her baby [23].

Despite its negative effects, in-depth information on prelacteal feeding remains scarce in Vietnam. To address this gap, this paper aims to document prelacteal feeding practices and to identify potential factors associated with prelacteal feeding among Vietnamese mothers. The findings generated by this study will help guide policies and actions concerned with increasing optimal infant feeding practices as a means for improving the nutrition and health of children.

## Methods

### Data sources and study population

Data were drawn from a household survey conducted in Vietnam under Alive & Thrive (A&T) in 2011. A&T is an initiative that aims to reduce undernutrition and death caused by suboptimal infant and young child feeding (IYCF) practices in Vietnam, Bangladesh, and Ethiopia. This cross-sectional survey was carried out in 11 provinces belonging to four out of Vietnam’s six ecological regions where A&T operates, including: Hanoi, Hai Phong, Quang Binh, Quang Tri, Da Nang, Quang Nam, Khanh Hoa, Dak Lak, Dak Nong, Tien Giang, and Ca Mau. A representative sample of pairs of mothers and their children under 24 months of age was selected by a three-stage cluster sampling technique: 1) selection of communes in program districts; 2) selection of primary sampling units based on the population-proportionate-to-size method; and 3) selection of mother–child pairs using systematic random sampling. A total of 11,046 mother-child pairs participated in the survey. Mothers were informed about the purpose of the study, and written informed consent was obtained from all participants. Data were collected via face-to-face interviews using a structured questionnaire. Because this paper focuses on the predictors of breastfeeding practices, analyses were restricted to the 6068 mother-child dyads in which the child was under six months old. Ethical approval was obtained from the Institutional Review Board of the Institute of Social and Medical Studies in Hanoi.

### Measures

#### Dependent variables

The main dependent variable was prelacteal feeding, defined as the administration of any foods or liquids other than breastmilk to an infant during the first three days after birth [9,24]. This definition is based on the knowledge that breastfeeding is established when the breasts begin to secrete copious amounts of milk, about four days after delivery [25]. In order to provide a more in-depth understanding of mother’s feeding choices, additional analyses were run to examine the following two outcomes: 1) prelacteal feeding of plain water and 2) prelacteal feeding of infant formula. These feeds were highlighted because they are the two most commonly fed prelacteals in Vietnam.

#### Independent variables

**Figure 1:**
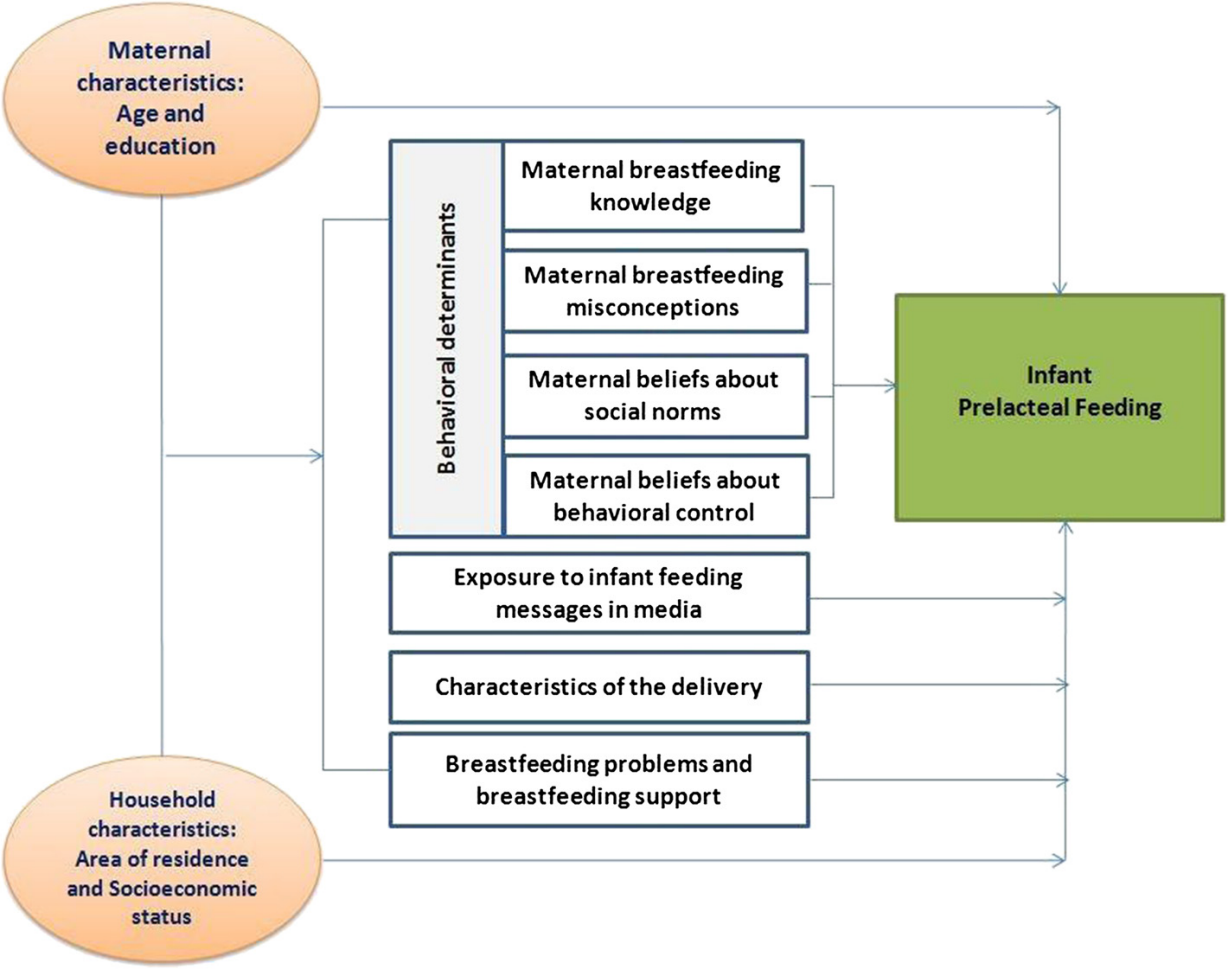
Conceptual framework of the determinant factors of infant prelacteal feeding practices.

The selection of potential determinants of prelacteal feeding was guided by the conceptual framework (Figure 1). These variables were divided into four behavioral determinant categories including maternal breastfeeding knowledge, conceptions, beliefs about social norms, and behavioral control. The other three determinant categories were exposure to infant feeding messages in the media, characteristics of the delivery, and breastfeeding problems and support. We controlled for maternal and household characteristics that may influence breastfeeding practices.

#### Maternal breastfeeding knowledge

Maternal breastfeeding knowledge was assessed based on mothers’ answers to three questions related to early breastfeeding: 1) “How long after birth should a newborn start breastfeeding?” (Correct: Immediately or less than one hour after delivery); 2) “What should a mother do with the ‘first milk’ or colostrum?” (Correct: Give it to the infant); and 3) “On average, how many milliliters (ml) of milk does a newborn need per feed on the first day after birth?” (Correct: 5–7 ml) [26]. Each question was given a score of 1 or 0 depending on a correct or incorrect response respectively. Due to the large number of knowledge and belief indicators, analyzing each variable separately could lead to multicollinearity or overfitting in the multivariate models [27]. We therefore generated an overall score of correct knowledge (0–3) in order to detect if the odds of prelacteal feeding changed as breastfeeding knowledge increased.

#### Maternal breastfeeding misconceptions

To assess whether they held common breastfeeding misconceptions, mothers were asked whether they agreed or disagreed with 10 beliefs about breastfeeding including: 1) “My breastfed infant under six months will be thirsty if not given water;” 2) “My breastfed newborn will be hungry if not given infant formula within 24 hours of birth;” 3) “EBF under six months does not provide all the nutrients needed for infant health;” 4) “Feeding a combination of breastmilk and infant formula under six months provides the best possible nutrition;” 5) “An infant will get thrush if his/her mouth is not cleaned with water after breastfeeding;” 6) “Good attachment to the breast is not needed for sufficient milk production;” 7) “Women with small breasts have difficulty producing enough breastmilk;” 8) “Being relaxed during breastfeeding does not help the body produce sufficient milk;” 9) “My body will produce the same amount of breastmilk even if I feed infant formula;” and 10) “EBF under six months does not provide all the nutrients needed for optimal brain development.” Mothers’ responses were used to assess whether they held these 10 common breastfeeding misconceptions. An overall scale representing the number of misconceptions a mother held (0–10) was created for analysis in the multivariate models. For ease of interpretation, these scores were then divided into tertiles for multivariate analyses: low (≤2 misconceptions), medium (3–4 misconceptions), and high (5–8 misconceptions).

#### Maternal beliefs about social norms

Women’s beliefs about the social norms surrounding EBF were assessed based on whether respondents agreed with the following statements: 1) “Most people whose opinions are important to me think that I should feed my infant only breastmilk and no other food, water, or infant formula for the first six months;” and 2) “Most women who have infants like me feed their infants only breastmilk and no other food, water, or infant formula for the first six months.” Agreeing with these statements was indicative of holding beliefs that the social norms favor EBF. Similar to breastfeeding knowledge, a scale representing the number of beliefs mothers’ held that favor EBF (0–2) was created for examination in the multivariate models.

#### Maternal beliefs about behavioral control

Four items were used to measure mothers’ beliefs about their own sense of control and self-efficacy over certain breastfeeding practices: 1) “My body can produce enough colostrum to feed my newborn within one hour of birth;” 2) “My body can produce enough breastmilk to feed my newborn only breastmilk in the first 24 hours after birth;” 3) “My body’s ‘first milk’ is all my newborn needs in the first 24 hours after birth;” and 4) “I can refuse to let my mother-in-law feed infant formula to my newborn in the first 24 hours after birth.” Mothers’ levels of confidence in relation to these statements were categorized as confident versus unconfident. In the logistic regression models, the items were combined to form a scale of the number of statements in which mothers felt confident about behavioral control (0–4).

#### Exposure to infant feeding messages in the media

Exposure to media was based on mothers’ reported exposure to one of the following messages on TV in the 30 days prior to interview: breastfeeding information, infant formula advertising, both of the above, or neither of the above.

#### Characteristics of the delivery

Delivery characteristics included self-reported mode of delivery for the index child (vaginal delivery with or without episiotomy or caesarean section (C-section)). We also examined whether the mother or her family brought infant formula to the health facility during delivery. Delivery place was not examined because only 2.9% of women delivered at home while all others delivered at a public or private health facility (94.8% and 2.3%, respectively).

#### Breastfeeding problems and breastfeeding support

Breastfeeding problems were defined as whether a mother had experienced one or more of the following: a belief that she has insufficient breastmilk, the infant has difficulty attaching or suckling, and/or pain, engorgement, or cracked nipples. Breastfeeding support was defined as breastfeeding counseling or advice given by a family member or health worker. Mothers were asked whether they had received this support during pregnancy and during the first three days after delivery.

#### Maternal and household characteristics

Maternal characteristics examined as control variables included age and education. Household-level variables included area of residence (rural or urban) and household socioeconomic status (SES). Household SES was calculated using a principal components analysis of asset data such as house and land ownership, housing quality, access to services (electricity, gas, water, and sanitation services) and household assets (productive assets, durable goods, animals, and livestock). The first component derived from component scores was used to divide household SES into quartiles [28,29]. We also adjusted for child sex in the analyses. Due to its high correlation with area of residence, maternal occupation (farmer or non-farmer) was excluded from analyses. In addition, ethnicity was not examined due to the small proportion of ethnic minorities in the sample (6.9%).

### Statistical analysis

Stata version 11 software was used to analyze the data [30]. Descriptive analyses were used to describe the prelacteal feeding practices and characteristics of the study population. Multivariate logistic regression models were applied to analyze the factors associated with prelacteal feeding outcomes controlling for other covariates as well as cluster effects at the commune level. The final models present the results as odds ratios (OR) with 95% confidence intervals (95% CI). All of the results were considered significant at p <0.05.

## Results

### Characteristics of participants

Background characteristics of the study population are presented in Table 1. The mean age of mothers was 27.7 years. Only 11.4% of women had attended primary school or less, while nearly half completed secondary school (48.2%), and the rest completed high school or higher education. The majority of the study population lived in rural areas (83.3%).

**Table 1:** Background characteristics of the study population and selected independent variables

**Background characteristics**	**Percent**	**Selected independent variables**	**Percent**
Maternal age (years)	27.3 ± 5.5*	Mode of delivery	
15–24	38.0	Vaginal delivery	37.4
25–29	34.3	Vaginal delivery + episiotomy	41.3
30–34	16.8	C-section	21.3
35–51	11.0	Brought infant formula to delivery room	21.3
Maternal education		Experienced breastfeeding problems	10.6
Primary school or less	11.4	Received breastfeeding support from a family member	
Secondary school	48.2	During pregnancy	31.8
High school	24.5	During first 3 days after delivery	31.9
College or higher	15.9	Received breastfeeding support from a health care provider	
Female child	48.0	During pregnancy	44.1
Rural area of residence	83.3	During first 3 days after delivery	31.6
SES in quartiles	25.0	Exposure to infant feeding messages on TV in past 30 days	
		Saw only breastfeeding information	2.6
		Saw only infant formula advertising	36.5
		Saw both infant formula and breastfeeding advertising	43.3
		Saw neither message	17.6

*Mean ± SD.

### Prelacteal feeding patterns

**Figure 2:**
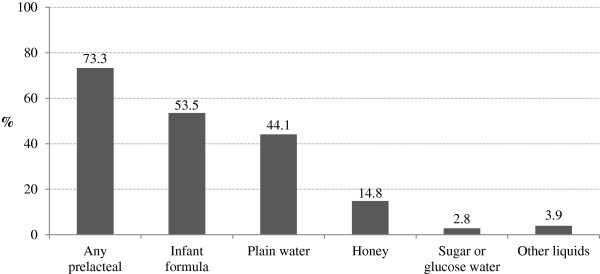
Prevalence of prelacteal feeding in the study population.

Prelacteal feeding was very common, practiced by nearly three out of four respondents (73.3%) (Figure 2). Infant formula was the most commonly fed prelacteal (53.5%), followed by plain water (44.1%). Other prelacteal feeds included honey, glucose water, and other liquids. Nearly all of the mothers in this study reported feeding colostrum to their infants (95.7%).

### Maternal breastfeeding knowledge

**Figure 3:**
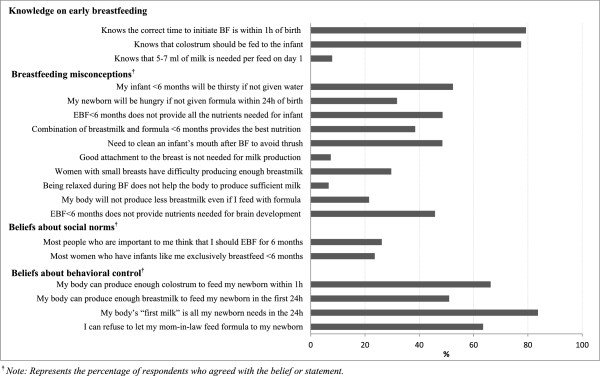
Selected behavioral determinants of infant feeding practices.

More than three-quarters of mothers knew that breastfeeding should begin within one hour of birth and that babies should be fed colostrum (Figure 3). However, only 7.9% of mothers knew that newborns only need 5–7 ml of milk per feed on the first day of life. In all three logistic regression models, higher breastfeeding knowledge scores correspond to lower odds of feeding prelacteals in general and feeding of plain water and infant formula specifically. For example, women with three correct answers had about half the odds of feeding any prelacteals (OR: 0.49, 95% CI: 0.32–0.75) compared to those who answered none of the knowledge questions correctly (Table 2).

**Table 2:** Multivariate logistic regression results of the factors associated with prelacteal feeding

**Independent variables**	**Any prelacteal**		**Plain water**		**Infant formula**	
	**OR**	**95% CI**	**OR**	**95% CI**	**OR**	**95% CI**
**Behavioral determinants**						
Number of correct breastfeeding knowledge (ref: 0)						
1	0.74	0.52–1.06	0.76	0.59–0.97	0.68	0.52–0.91
2	0.62	0.44–0.88	0.66	0.52–0.83	0.65	0.49–0.85
3	0.49	0.32–0.75	0.50	0.35–0.70	0.42	0.29–0.62
Level of breastfeeding misconceptions (ref: Low)						
Medium	2.01	1.68–2.42	1.86	1.61–2.15	1.52	1.29–1.80
High	2.09	1.74–2.50	1.95	1.69–2.24	1.85	1.58–2.17
Number of beliefs that social norms favor EBF (ref: 0)						
1	0.65	0.53–0.78	0.52	0.44–0.62	0.93	0.77–1.12
2	0.45	0.38–0.53	0.30	0.25–0.36	0.86	0.72–1.02
Number of beliefs favoring behavioral control (ref: 0)						
1	0.48	0.28–0.82	0.89	0.67–1.18	0.63	0.43–0.90
2	0.43	0.25–0.71	0.87	0.67–1.14	0.43	0.30–0.60
3	0.24	0.14–0.39	0.83	0.63–1.07	0.23	0.16–0.32
4	0.12	0.07–0.19	0.55	0.42–0.73	0.12	0.09–0.17
**Exposure to media**						
Exposure to infant feeding messages on TV in past 30 days (ref: Saw only breastfeeding information)						
Saw only infant formula advertising	1.05	0.69–1.59	1.11	0.75–1.63	1.10	0.73–1.66
Saw both infant formula advertising and breastfeeding information	1.07	0.72–1.61	1.26	0.86–1.84	0.94	0.63–1.40
Saw neither message	1.33	0.89–1.99	1.25	0.86–1.81	1.12	0.75–1.65
**Characteristics of the delivery**						
Mode of delivery (ref: Vaginal delivery without episiotomy)						
Vaginal delivery + episiotomy	1.36	1.17–1.58	1.16	1.02–1.32	1.77	1.54–2.04
Caesarean section	2.94	2.39–3.61	1.24	1.06–1.45	5.64	4.69–6.78
Brought formula to delivery room (ref: No)	2.39	1.96–2.92	0.98	0.86–1.13	3.77	3.19–4.46
**Experienced breastfeeding problems** (ref: No)	1.31	1.04–1.66	1.00	0.83–1.20	1.48	1.21–1.82
**Received breastfeeding support**						
From a family member (ref: no support)						
During pregnancy	0.92	0.79–1.07	1.01	0.89–1.15	0.91	0.79–1.05
During first 3 days after delivery	1.15	0.98–1.34	1.14	1.00–1.30	0.95	0.82–1.10
From a health care provider (ref: no support)						
During pregnancy	0.81	0.71–0.93	1.10	0.98–1.24	0.82	0.72–0.93
During first 3 days after delivery	0.84	0.73–0.97	0.98	0.87–1.12	0.85	0.74–0.98
**Maternal characteristics**						
Age (years) (ref: 15–24)						
25–29	1.01	0.86–1.19	1.08	0.94–1.24	1.05	0.90–1.22
30–34	0.85	0.70–1.04	1.01	0.85–1.20	1.00	0.82–1.21
35–51	1.00	0.79–1.26	1.24	1.01–1.52	0.96	0.77–1.20
Education (ref: Primary school or less)						
Secondary school	1.01	0.82–1.26	0.78	0.64–0.93	1.19	0.97–1.46
High school	1.04	0.81–1.33	0.65	0.52–0.80	1.35	1.07–1.70
College or higher	0.94	0.70–1.26	0.57	0.44–0.73	1.41	1.08–1.86
Female child (ref: Male)	1.02	0.90–1.17	1.07	0.96–1.20	1.03	0.91–1.17
**Household characteristics**						
Urban area of residence (ref: Rural)	2.11	1.68–2.65	2.92	2.49–3.43	1.72	1.44–2.06
Household SES (ref: Lowest quartile)						
Middle-low quartile	1.34	1.12–1.62	1.25	1.06–1.48	1.64	1.37–1.96
Middle-high quartile	1.47	1.21–1.78	1.06	0.90–1.25	2.25	1.87–2.70
Highest quartile	1.98	1.60–2.46	1.06	0.88–1.27	3.31	2.71–4.06

### Maternal breastfeeding misconceptions

Breastfeeding misconceptions were widespread in the study population (Figure 3). The most commonly held misconceptions were: breastfed infants will be thirsty if not given water before six months (52.4%); EBF in the first six months does not provide all the nutrients needed for infant health (48.6%) or for optimal brain development (45.8%); and an infant’s mouth should be cleaned with water after breastfeeding in order to prevent thrush (48.5%). Logistic regression analyses indicate that the more misconceptions a mother held, the higher her odds of feeding prelacteals (Table 2). Compared to mothers with the fewest misconceptions (≤2), those who held the most misconceptions (5–8) had about twice the odds of giving prelacteals (OR: 2.09, 95% CI: 1.74–2.50). This group had the greatest odds of practicing prelacteal feeding of both water and infant formula.

### Maternal beliefs about social norms

Only one-quarter of the study population perceived that EBF in the first six months is a social norm in their communities (Figure 3). In the multivariate models, women who believed social norms favor EBF had lower odds of feeding prelacteals in general (OR: 0.45, 95% CI: 0.38–0.53) and water specifically (OR: 0.30, 95% CI: 0.25–0.36). However, the association is not significant in the model for prelacteal feeding of infant formula (OR: 0.86, 95% CI: 0.72–1.02) (Table 2).

### Maternal beliefs about behavioral control

Roughly two out of three mothers were confident that they could produce enough colostrum to feed their newborn within one hour of birth, and 83.7% were confident that their ‘first milk’ was all that was needed in the first 24 hours (Figure 3). However, about half of mothers lacked confidence in their ability to produce enough breastmilk to exclusively breastfeed for the first 24 hours. Mothers reporting the highest levels of confidence in behavioral control had the lowest odds of feeding water, infant formula, and other prelacteals to their newborns (Table 2).

### Exposure to infant feeding messages in the media

While 79.8% of respondents had seen infant formula advertising, only 45.9% had seen breastfeeding information on TV in the past 30 days (Table 1). There was a large overlap as 43.3% of mothers had seen both infant formula advertising and breastfeeding promotion on TV during that period. Bivariate analyses show that mothers who had only seen infant formula advertising were the most likely to feed any prelacteals (including both water and infant formula), while mothers who had only seen breastfeeding information on TV were the least likely to do so. However, the association between media exposure and prelacteal feeding practices was not statistically significant in the logistic regression models (Table 2).

### Characteristics of the delivery

The most common mode of delivery was vaginal delivery with episiotomy (41.3%), followed by vaginal delivery without episiotomy (37.4%) (Table 1). Roughly one-fifth of mothers delivered by C-section. Mode of delivery was strongly associated with prelacteal feeding behaviors (Table 2). Compared with respondents who delivered vaginally without an episiotomy, mothers who delivered by C-section had more than five times the odds of feeding infant formula to their newborns (OR: 5.64, 95% CI: 4.69–6.78), and those who delivered vaginally with episiotomy had nearly twice the odds of doing so (OR: 1.77, 95% CI: 1.54–2.04) (Table 2).

About one-fifth of the sample brought infant formula with them to the health facility for delivery, and 80.2% of those mothers fed infant formula to their newborns. Even among mothers who did not initially bring formula with them to the facility, close to half (46.3%) obtained infant formula after delivery and fed it to their newborns. Bringing infant formula to the health facility was associated with increased odds of formula feeding by nearly four times (OR: 3.77, 95% CI: 3.19–4.46) (Table 2).

### Breastfeeding problems and breastfeeding support

Breastfeeding problems were reported by 10.6% of mothers (Table 1). Compared to their counterparts who reported no problems, the odds were higher that these mothers fed prelacteals in general and infant formula specifically (Table 2). Only one-third of mothers reported receiving breastfeeding support from a family member while pregnant or during the three days following delivery, and less than half received breastfeeding counseling or advice from a health care provider during these critical periods (Table 1). Health worker-delivered breastfeeding support was associated with lower odds of prelacteal feeding in general and infant formula specifically, and this association was found when support was provided both during pregnancy and early postpartum (Table 2). In contrast, mothers who received advice from family members soon after delivery had slightly increased odds of feeding water to their newborns.

### Maternal and household characteristics

As mothers’ level of education increased, their odds of feeding water declined while their odds of feeding infant formula increased (Table 2). There was also a trend in which the higher the household SES, the greater the odds that newborns were given prelacteals in general and formula specifically (Table 2). Compared to those in the lowest SES quartile, mothers in the highest quartile had about three times the odds (OR: 3.31, 95% CI: 2.71–4.06) of feeding infant formula to their newborns (Table 2). In addition, area of residence was strongly associated with prelacteal feeding practices. Mothers who lived in urban areas had triple the odds of feeding water (OR: 2.92, 95% CI: 2.49–3.43) and almost double the odds of feeding infant formula as prelacteals (OR: 1.72, 95% CI: 1.44–2.06) (Table 2).

## Discussion

While surveillance surveys show that early initiation of breastfeeding is relatively high in Vietnam (62%) [1], our data indicate that prelacteal feeding is highly prevalent (73.3%). This practice not only interferes with exclusive breastfeeding in the first few days after birth, but also with optimal breastfeeding practices thereafter. In another paper (publication in progress), analyses of the same data found that prelacteal feeding was negatively associated with EBF in the first six months of life. Despite its serious implications for child nutrition, prelacteal feeding is not explicitly tracked by the current health system and is often overlooked as a consequence.

Multiple factors were found to predict the prelacteal feeding practices of Vietnamese mothers. Among those, the most highly modifiable risk factors were incorrect breastfeeding knowledge, breastfeeding misconceptions, and beliefs about social norms and behavioral control related to breastfeeding. Previous studies have reported that some Vietnamese mothers avoid feeding colostrum out of fear that it makes infants sick, brings ‘bad luck,’ or holds no nutritional value [31,32]. However, maternal knowledge of the need for colostrum feeding and the timing of breastfeeding initiation were moderately high in this study population, suggesting that mothers primarily feed prelacteals for reasons other than colostrum avoidance. Few participants knew that newborns need only 5–7 ml of milk per feed on the first day, which may partially explain why Vietnamese mothers commonly feel that they have insufficient breastmilk [12,33] and thus supplement breastfeeding with prelacteals [32].

Our findings also confirm previous research showing that breastfeeding misconceptions are highly prevalent and strongly associated with increased likelihood of prelacteal feeding [12,32]. Further, mothers who believed that social norms favor EBF and those who were confident in their ability to follow optimal breastfeeding practices had lower odds of prelacteal feeding. These findings highlight an urgent need for developing behavior change communication interventions that improve breastfeeding knowledge, beliefs, and social norms. To prevent prelacteal feeding, programs should focus on helping mothers adopt beliefs that they can produce enough colostrum and breastmilk to feed infants in the first 24 hours of life. These programs should also tackle the norm of early water feeding by emphasizing that breastmilk contains all the water and nutrients a baby needs to be healthy, both immediately following birth and in the six months thereafter.

Among the behavior change communication strategies, individual or group counseling is one of the most effective for increasing rates of optimal breastfeeding [6][34]. This study offers further evidence that provider-delivered breastfeeding counseling during pregnancy and postpartum may discourage prelacteal feeding. Despite the benefits, only one-third of participants received breastfeeding support from health staff during these critical periods. Although Vietnam implemented the Baby-Friendly Hospital Initiative (BFHI) in 1993, breastfeeding counseling services for pregnant women and lactating mothers are lacking [35]. Barriers to these services include inadequate training, poor counseling skills, low confidence, and limited understanding of breastfeeding among health workers [12,33,34]. Providers should be monitored and given support to ensure compliance with BFHI guidelines, and emphasis should be placed on integrating breastfeeding counseling into routine antenatal and postpartum care.

Consistent with previous research [9,36], we found that high rates of C-section and episiotomy may contribute to high rates of prelacteal feeding. The link between mode of delivery and feeding practices may be explained in part by mothers’ common concern that the antibiotics that they receive during their care could harm their infants, which discourages them from breastfeeding [31,37]. In addition, the pain and discomfort associated with C-section and episiotomy procedures has been reported to delay the initiation of breastfeeding [37,38].

Considerable evidence demonstrates that peer counseling and social support are highly effective in improving breastfeeding practices [39], particularly programs that feature community IYCF educators and home visits [40,41]. These interventions are especially well suited for Vietnam, where volunteers can be drawn from the extensive village health worker network in the Government health care system or from the Women’s Union which has members across the county. A major advantage to these programs is their potential to reach not only pregnant women and mothers, but also their families. Family members have been shown to have considerable influence over the breastfeeding practices of Vietnamese mothers [12,31,33], and our findings show that family members may play a role in encouraging the early feeding of water. Thus, educating families as a whole has a high potential to positively shape mothers’ IYCF beliefs and practices.

Infant formula marketing has been found to adversely affect breastfeeding beliefs and behaviors in Vietnam [12,33,38], and our bivariate results provide some evidence that this marketing encourages prelacteal feeding. We posit that the relationship disappears in the multivariate models due to the inclusion of breastfeeding beliefs covariates. In models run without these variables, exposure to infant formula advertising was significantly associated with increased odds of feeding prelacteals (OR: 1.64, 95% CI: 1.13–2.37). This advertising therefore appears to affect prelacteal feeding by shaping breastfeeding beliefs. To combat this influence, Vietnam’s Decree 21/2006/ND-CP (Decree 21) regulates the marketing of nutrition products for children, including infant formula. However, a 2011 review found that the law’s effectiveness is severely hampered by its weak enforcement and compliance, allowing companies to regularly violate the law and exploit its loopholes [42]. In the face of this challenge, it is heartening to find evidence in our study that the effects of infant formula advertising on prelacteal feeding were diluted by exposure to breastfeeding information on TV. It is recommended that efforts are made to strengthen the monitoring and enforcement of Decree 21 and that these efforts are accompanied by mass media campaigns that promote breastfeeding and challenge some of the messages conveyed by infant formula advertising.

One-fifth of mothers brought infant formula to their delivery, and these mothers were significantly more likely to engage in prelacteal feeding. Also of concern, however, is the relatively large proportion of mothers who did not bring infant formula with them to the health facility but still fed the product to their newborns. This result highlights the ease of accessibility of infant formula near or within health facilities. While Decree 21 prohibits the sale of formula in health facilities, the law allows for their sale in the pharmacies of these facilities [42]. Also problematic are reports that formula companies provide health facilities and staff with incentives such as donations of supplies, scholarships, workshops, and even sales commissions, a violation of the law [42,43]. Health facilities should be regularly inspected for compliance with Decree 21, and leaders of these facilities must commit to eradicating the promotion of infant formula products on their premises.

This study contains some methodological limitations that deserve consideration. First, its cross-sectional design limits conclusions to be drawn about the causality. Second, because mothers were asked to retrospectively report their feeding practices during the first three days following delivery, we must allow for the possibility of recall bias. However, there is little reason to believe that mothers differentially recalled information based on their exposure and outcome status, reducing the likelihood that recall bias significantly influenced our results. Third, there were insufficient data to account for cases in which supplementary feeding of newborns may be justified such as among infants who are very preterm, infants with very low birth weight, and mothers living with HIV or severe illness [44]. Because these conditions affect very few mothers and infants, this constraint should not greatly alter our results nor the conclusions drawn. Despite these limitations, the current study is the first to provide an in-depth examination of prelacteal feeding in a large, diverse population in Vietnam and has important implications for future breastfeeding promotion interventions in the region.

## Conclusions

The predominance of risky prelacteal feeding behaviors stresses the need for nutrition interventions in Vietnam that focus on improving breastfeeding practices during the first days of an infant’s life. Eradicating a deeply-rooted norm such as prelacteal feeding requires a multi-level and multi-sectoral strategy that incorporates training, interpersonal communication, mass media messages, community activities, and legislative action. To give mothers sufficient support to establish and continue optimal breastfeeding practices, it is critical to reach them as early as possible – during pregnancy and immediately after giving birth. Most importantly, efforts are needed to ensure that health facilities offer supportive environments for early and exclusive breastfeeding, including avoidance of prelacteal feeds. These activities should be supported by mass media campaigns and community-level actions such as educational programs that enhance IYCF knowledge and dispel common breastfeeding myths. Community health volunteers should also be leveraged to educate not only mothers, but also their family members on the benefits of breastfeeding and the potential harm of prelacteal feeding. Together, these efforts have significant potential to improve breastfeeding practices in Vietnam.

## Competing interests

The authors declare that they have no competing interests.

## Authors’ contributions

PHN contributed to developing the study design and research questions, conducting the statistical analysis of data, and drafting and revising the manuscript. SCK contributed to conducting the literature review, providing input, and drafting, editing and revising the manuscript. NTN contributed to developing study design and providing input and comments on the manuscript. TTN contributed to developing the study design, interpreting the data and providing input and comments on the manuscript. LMT assisted in data analyses and preparation of the tables and figures. NH contributed to developing the study design and provided input and comments for the manuscript. All authors contributed in the development, review and approval of the final manuscript.

## Pre-publication history

The pre-publication history for this paper can be accessed here:

http://www.biomedcentral.com/1471-2458/13/932/prepub
